# A Novel Subtype of Acquired Generalized Lipodystrophy Associated With Subcutaneous Panniculitis-Like T-cell Lymphoma

**DOI:** 10.1210/jcemcr/luae069

**Published:** 2024-04-29

**Authors:** Fieke W Hoff, Chao Xing, Abhimanyu Garg

**Affiliations:** Department of Internal Medicine, University of Texas Southwestern Medical Center, Dallas, TX 75390, USA; McDermott Center for Human Growth and Development, Department of Bioinformatics, O’Donnell School of Public Health, University of Texas Southwestern Medical Center, Dallas, TX 75390, USA; Department of Internal Medicine, University of Texas Southwestern Medical Center, Dallas, TX 75390, USA; Section of Nutrition and Metabolic Diseases, Division of Endocrinology, Department of Internal Medicine and the Center for Human Nutrition, University of Texas Southwestern Medical Center, Dallas, TX 75390, USA

**Keywords:** acquired generalized lipodystrophy, subcutaneous panniculitis-like T-cell lymphoma, hemophagocytic lymphohistiocytosis

## Abstract

Acquired generalized lipodystrophy (AGL) is an extremely rare disease that is characterized by loss of body fat affecting nearly all parts of the body. It is often associated with autoimmune diseases or panniculitis, whereas in other patients the underlying etiology is unclear. We report a 52-year-old male individual who was diagnosed with subcutaneous panniculitis-like T-cell lymphoma (SPTCL) that spontaneously went into remission. Years later he developed new subcutaneous nodules most concerning for relapse SPTCL or lupus panniculitis, followed by onset of hemophagocytic lymphohistiocytosis (HLH) that was treated with allogeneic stem cell transplantation. Notably, around the same time, he also developed generalized subcutaneous fat loss of both upper and lower extremities, chest, abdomen, and face that persisted after treatment of the HLH. Whole exome sequencing was performed to search for pathogenic variants that are associated with SPTCL, including those in hepatitis A virus cellular receptor 2 (*HAVCR2*), but did not detect any potential disease-causing variant. Our report brings to the attention a novel subtype of panniculitis-variety of AGL. Whether generalized loss of subcutaneous fat in this patient is due to lymphoma-associated panniculitis or due to development of adipose tissue-directed autoantibodies as a paraneoplastic “autoimmune” manifestation of SPTCL remains unclear.

## Introduction

Lipodystrophies are a group of rare, heterogeneous disorders characterized by selective loss of body fat in the absence of nutritional deprivation, which can be generalized (involving nearly all parts of the body), partial (affecting the limbs or upper body), or localized (affecting small areas or regions) (1‐4). In some patients, the loss of fat is autoimmune-mediated and affects nearly all the regions of the body, called *acquired generalized lipodystrophy* (AGL). AGL is an ultra-rare disorder with only ˜100 cases reported so far in the literature (4). Most of the patients with AGL have an associated autoimmune disease (eg, juvenile dermatomyositis, systemic lupus erythematosus, rheumatoid arthritis) or panniculitis (presenting as subcutaneous nodules characterized pathologically by infiltration of adipose tissue with lymphocytes, histiocytes, and multinucleated giant cells), while in others the underlying mechanism is unclear (idiopathic) (5). Recently, anti-perilipin 1 (PLIN1) autoantibodies have been reported in patients with AGL, especially in those with the panniculitis-variety (6‐8). We report a novel subtype of panniculitis-variety of AGL associated with subcutaneous panniculitis-like T-cell lymphoma (SPTCL) and hemophagocytic lymphohistiocytosis (HLH).

## Case Presentation

A 52-year-old African American man had SPTCL diagnosed at the age of 34 years that went into remission without any treatment. Twelve years later, he developed multiple subcutaneous nodules distributed all over the body as well as generalized subcutaneous fat loss, concerning for relapsed SPTCL. Skin punch biopsies were taken from the right chest and right forearm, showing lymphoplasmacytic infiltrate primarily involving the subcutis with some extension into the deep dermis and periadnexal fat with abundant mucin deposition. Lymphocytes were CD3+ and CD5+ with a subpopulation of CD8+ and CD4+ T-cells. Staining was positive for beta F1 and most of the CD8+ cells were expressing TIA-1. Stainings for CD30, CD25, and TCR gamma were negative. While findings were largely indeterminate, they were most consistent with SPTCL or lupus panniculitis.

During the following month, he gradually became sicker, including symptoms of daily fevers, weight loss, night sweats, anorexia, anasarca, confusion, and generalized muscle atrophy and lipodystrophy. Laboratory studies showed bi-lineage cytopenias, as well as abnormal liver function tests, hyperferritinemia, hypertriglyceridemia, hypofibrinogenemia, hypoalbuminemia, and an elevated D-dimer. Serum inflammatory markers were elevated. An extensive infectious, autoimmune, and malignancy workup was negative, including absence of malignant cells in the cerebrospinal fluid. Positron emission tomography and computerized tomography scans showed nonspecific metabolically active enlarged axillary and inguinal nodes and a normal sized spleen (12.7 cm). Bilateral bone marrow biopsies revealed a hypercellular bone marrow (∼90%) with an atypical polymorphous lymphohistiocytic infiltrate admixed with eosinophils and plasma cells, scattered hemophagocytosis, and no evidence of lymphoma involvement.

His overall presentation was most consistent with HLH, and there was concern for potential relapse of SPTCL, as HLH can precede fulminant T-cell lymphoma relapse and there is a strong association between SPTCL and HLH (9, 10). He was treated per HLH-94 protocol, comprising weekly treatments with etoposide and dexamethasone (10). While he initially showed good response to treatment, the disease subsequently progressed. Treatment was switched to 2 cycles of tocilizumab combined with etoposide resulting in partial response, followed by an allogeneic hematopoietic stem cell transplant (HSCT) from an HLA-matched related donor (sibling). He achieved sustained complete remission of both SPTCL and HLH. Graft-vs-host disease (GVHD) prophylaxis included tacrolimus and mycophenolate mofetil. His post-HSCT course was complicated by moderate chronic GVHD of the skin, mouth, and eyes.

**Figure 1.**
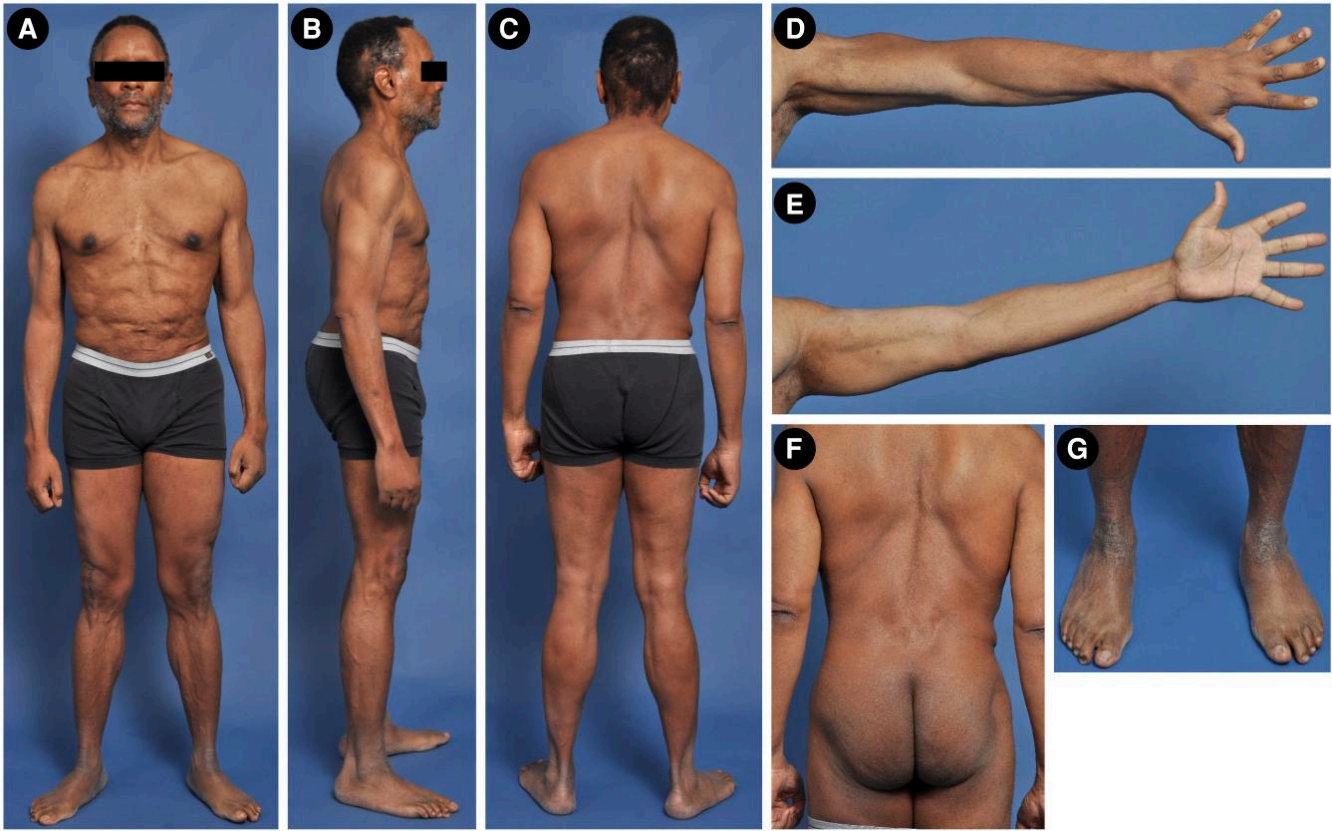
Clinical features of the 52-year-old African American man with acquired generalized lipodystrophy due to subcutaneous panniculitis-like T-cell lymphoma. A, Anterior; B, Right lateral; and C, Posterior view of the patient, showing temporal recession of hair, marked loss of fat from the temporal region and cheeks, from the neck, thorax, abdomen, upper extremities, thighs, and calves resulting in prominent musculature. There is focal loss of hair on the right temporal region and his eyebrows. D, Posterior; and E, Anterior view of the left upper extremity, showing loss of subcutaneous fat resulting in prominence of muscles. There was a hyperpigmented macular lesion of about 3 cm diameter on the dorsum of the left hand. F, Posterior view of the trunk showing prominent gluteal muscles due to loss of subcutaneous fat. G, Anterior view of the calf and feet showing increased pigmentation of the skin and loss of subcutaneous fat from the dorsum of the feet.

**Figure 2.**
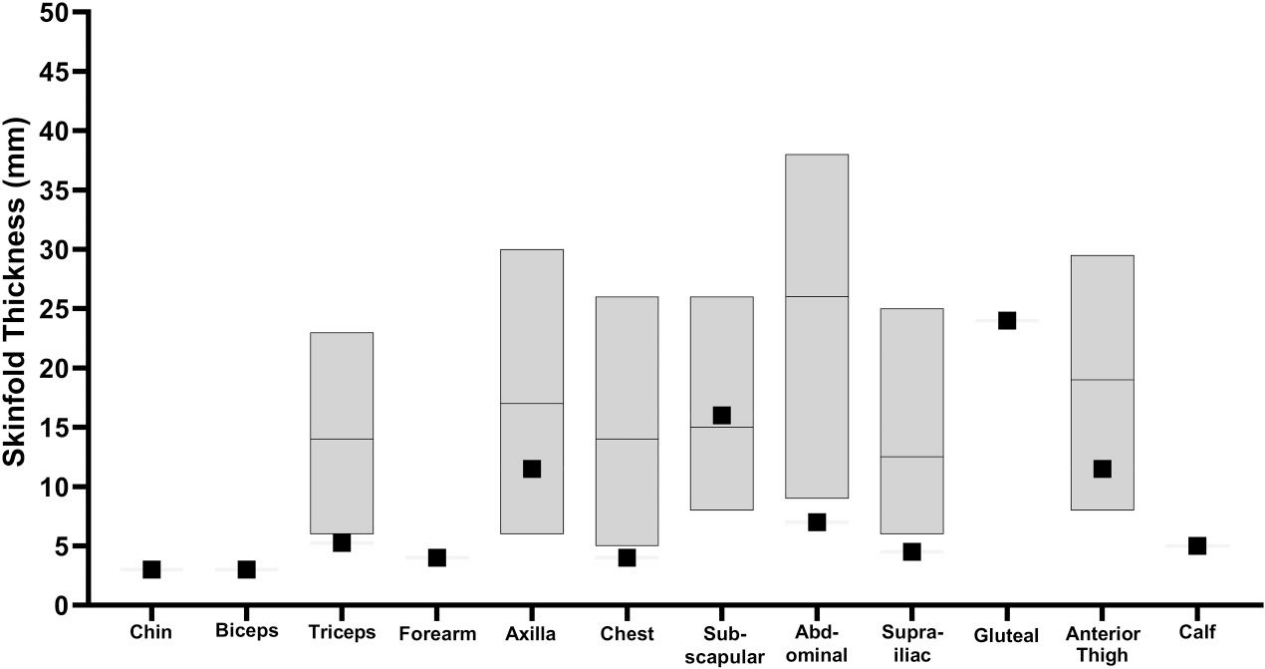
Skin fold thickness measurements of the patient compared to controls. Left to right: chin, biceps, triceps, forearm, axilla, chest, subscapular, abdominal, supra-iliac, gluteal, anterior thigh, calf. Black squares show the average value of 3 measurements for skinfold thickness of the patient (mm). The gray bars show normal range (10th-90th percentile) with the horizontal line in between showing the median value (*Jackson* et al. *Br J Nutr. 1978;40(3):497-504*).

**Figure 3.**
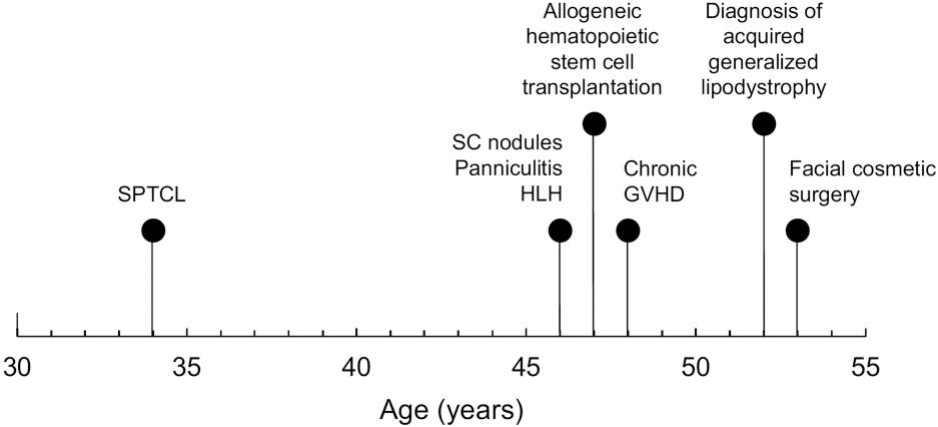
Chronological timeline of the different medical diagnoses. Abbreviations: GVHD, graft-vs-host disease; HLH, hemophagocytic lymphohistiocytosis; SC, subcutaneous; SPTCL, subcutaneous panniculitis-like T-cell lymphoma.

Four years after undergoing allogeneic HSCT, he presented for further evaluation of persistent subcutaneous fat loss that had started 6 years prior around the time of onset of the HLH. His body mass index (BMI) was 24.5 kg/m^2^ (height 174.0 cm, weight 75.5 kg). He was noted to have marked loss of subcutaneous fat from the temporal and malar regions bilaterally, as well as significant loss of fat from the anterior chest and abdomen (Fig. 1). There was mild midface hypoplasia, dermatochalasis of the facial skin, and dyschromia. His pectoralis major muscles appeared to be prominent. Fat loss from the calves was more apparent than it was at the bilateral thighs, and fat loss from the right hip was more compared to the left hip. He also had subcutaneous fat loss from the upper extremities. No pictures dated from before the onset of his fat loss were available for comparison. Skinfold thickness measurements revealed subcutaneous fat below the 10th percentile of normal values from several places over the body (Fig. 2). He had axillary acanthosis nigricans, and pigmentation on the tongue and nails as a result of his chronic GVHD. There were no xanthelasma, xanthomas, or hepato-splenomegaly. A chronologic timeline of the onset of the different clinical conditions is shown in Fig. 3.

## Diagnostic Assessment

Laboratory values showed total cholesterol of 102 mg/dL (2.64 mmol/L) (normal: < 200 mg/dL or 5.18 mmol/L), high-density lipoprotein (HDL)-cholesterol of 41 mg/dL (1.06 mmol/L) (normal: ≥ 40 mg/dL or 1.04 mmol/L), triglycerides of 137 mg/dL (1.55 mmol/L) (normal: ≤ 150 mg/dL or 1.69 mmol/L), fasting glucose level of 86 mg/dL (4.77 mmol/L) (normal: 65-99 mg/dL or 3.61-5.49 mmol/L), hemoglobin A1c of 5.9% (41.0 mmol/mol) (normal: < 5.7% or 38.8 mmol/mol), and an elevated insulin level of 21.9 µIU/mL (152.1 pmol/L) (normal: < 19.6 µIU/mL or 136.12 pmol/L). Notably, fasting serum triglycerides were elevated at 294 mg/dL and HDL-cholesterol was low at 32 mg/dL, a month prior. His basic metabolic panel and liver panel were within normal limits as was leptin level of 6.3 ng/mL (6.3 µg/L) (normal reference range for adult males with BMI range 18-25 kg/m^2^ is 0.3-13.4 ng/mL or 0.3-13.4 µg/L). His parents were both healthy, non-consanguineous, and he had 5 healthy siblings.

His DNA was isolated from buccal cells using the Easy-DNA kit (Invitrogen, Carlsbad, CA) and underwent whole exome sequencing using the Integrated DNA Technologies xGen Exome Research Panel V.1.0 on the Illumina platform. The mean coverage of the targeted regions was 97-fold with 89% bases covered by > 50-fold reads. Sequencing read length was paired-end 2 × 150 bp. Sequences were aligned to the human reference genome b37, and variants were called using the Genome Analysis Toolkit (11) and annotated using SnpEff. (12)

As recent studies have revealed germline biallelic pathogenic variants in hepatitis A virus cellular receptor 2 (*HAVCR2*) in SPTCL associated with HLH (13‐16), we searched for a homozygous or compound heterozygous disease-causing variants in *HAVCR2* and other genes that are likely to be associated with SPTCL: *ASXL1, BAZ2A, BRD2, CAPN1, JAK3, KMT2C, KMT2D, NUP98, PIAS3, PIK3CD, PLCG2,* and *UNC13D* (14). Besides, we performed exome-wide search for rare missense, nonsense, splicing, or frameshift variants homozygous in the proband. Other criteria included the minor allele frequency less than 0.01 in the genome aggregation database (gnomAD; http://gnomad.broadinstitute.org/), the Genomic Evolutionary Rate Profiling (GERP)++ score (17) greater than 2.0, and the Combined Annotation Dependent Depletion (CADD) score (18) greater than 15. HMZDelFinder was run to detect homozygous deletions (19).

We did not identify a potential disease-causing variant in the 13 candidate genes. No homozygous variant meeting the filtering criteria across the exome was found, nor was any pathogenic variant documented in ClinVar.

## Treatment

The general approach to treat AGL is to consume a low-fat diet and to treat metabolic disturbances (eg, hyperglycemia, hypertriglyceridemia). The patient maintained a healthy lifestyle, including regular physical activity and a balanced diet.

## Outcome and Follow-Up

Given the degree of facial disfigurement, he underwent cosmetic bilateral facial malar and temporal fossa expanded polytetrafluoroethylene implants with dermal fillers.

## Discussion

Patients with panniculitis-variety of AGL may have different underlying etiologies, which are not well understood at this time. First, many patients have associated autoimmune diseases, which suggests that the etiology is likely related to autoimmunity or an immune-mediated process (4). In support of underlying autoimmunity, PLIN1 autoantibodies were recently detected in about two-fifths of the patients with AGL, especially in those with panniculitis or autoimmune diseases (6‐8). However, whether serum PLIN1 autoantibodies are pathogenic and cause lipodystrophy or are just a marker of panniculitis-associated AGL, remains unclear at this time. Second, some patients have been reported to have reduced levels of serum complement 4 (20), which further suggests an autoimmune etiology. Here, we report a novel association of panniculitis-variety of AGL with a very unique subtype of lymphoma, SPTCL.

SPTCL is a rare subtype of peripheral T-cell lymphoma (PTCL) that localizes in the subcutaneous tissue and accounts for < 1% of all non-Hodgkin lymphomas (21, 22). It is characterized by CD8+ T-cells infiltrating into the subcutaneous adipose tissue, which constitutes panniculitis. The precise pathological differences among panniculitis in SPTCL and in other patients with panniculitis-associated AGL remains unclear. Remarkably, our patient, at his initial presentation with SPTCL, did not develop loss of subcutaneous fat or lipodystrophy, but developed panniculitis-associated AGL later with the onset of HLH. Thus, it is possible that at that time the pathology of the SPTCL changed and he started losing subcutaneous fat. Unfortunately, no skin biopsy was performed at the time of presentation with HLH and AGL.

Recent genetic studies have revealed recurrent biallelic germline pathogenic variants in *HAVCR2*, in 25% to 85% of the patients with SPTCL, that are associated with development of HLH (13‐16). *HAVCR2* encodes for a critical checkpoint molecule that regulates inflammatory responses (23). Our patient did not have any pathogenic variants in *HAVCR2* or other previously identified pathogenic gene variants associated with SPTCL.

AGL is considered a risk factor for PTCL, due to the link with autoimmunity (24, 25). However, a large case series (n = 76) by Misra and Garg, which included a review of the literature, did not report any association with lymphoma (5). Previous investigators reported 5 cases of lymphoma in patients with AGL, of which 3 patients developed lymphoma during metreleptin replacement therapy (25). However, 2 other patients developed lymphoma without metreleptin treatment (25). While the significance remains unclear, metreleptin has been reported to potentially contribute to development of T-cell lymphoma. Yet, our patient developed lymphoma first and AGL later.

Our literature search revealed only one case of a 46-year-old Caucasian man with Down syndrome who developed PTCL, followed by progressive lipodystrophy, new onset of diabetes mellitus with severe insulin resistance, hyperphagia, and hepatosplenomegaly consistent with the diagnosis of AGL 18 months later (26). Unlike our patient, this patient's skin biopsy did not reveal any panniculitis-like lesions. Esfandiari et al (27) reported a 33-year-old Caucasian female who developed SPTCL complicated by HLH. She was treated with 6 cycles of chemotherapy but relapsed and received multiple salvage regimens resulting in complete remission followed by allogeneic HSCT with no recurrence of the disease (27). She was then noted to have AGL with hypertriglyceridemia, hypoleptinemia, diabetes mellitus, and secondary amenorrhea. Interestingly, her fat loss started prior to the diagnosis of lymphoma, unlike our patient who developed AGL after the diagnosis of SPTCL.

In conclusion, we report a novel subtype of panniculitis-associated AGL in an adult with SPTCL who later developed HLH and underwent HSCT followed by GVHD. Whether generalized loss of subcutaneous fat in this patient is due to lymphoma-associated panniculitis or due to development of adipose tissue-directed autoantibodies as a paraneoplastic “autoimmune” manifestation of SPTCL remains unclear.

## Learning Points

- Acquired generalized lipodystrophy (AGL) is an extremely rare disease that is characterized by loss of body fat affecting nearly all parts of the body.
- AGL can be associated with an autoimmune disease or panniculitis, but the underlying etiology is often idiopathic.
- We present a novel subtype of panniculitis-variety of AGL associated with subcutaneous panniculitis-like T-cell lymphoma (SPTCL) and hemophagocytic lymphohistiocytosis (HLH).

## Data Availability

All datasets generated during and/or analyzed during the current study are not publicly available but are available from the corresponding author on reasonable request.

## Abbreviations

AGL: acquired generalized lipodystrophy
GVHD: graft-vs-host disease
HLH: hemophagocytic lymphohistiocytosis
HSCT: hematopoietic stem cell transplant
PTCL: peripheral T-cell lymphoma
SPTCL: subcutaneous panniculitis-like T-cell lymphoma
